# A Rare Case of Intracranial Extension of Ethmoidal Sinus Juvenile Psammomatoid Ossifying Fibroma

**DOI:** 10.1055/a-2779-6238

**Published:** 2026-01-21

**Authors:** Sagar Gawali, Shubham Goyal, Naren Nayak

**Affiliations:** 1 Department of Neurosurgery, L. T. M. Medical College and Sion General Hospital, Mumbai, Maharashtra, India

**Keywords:** fibro-osseous lesions, juvenile ossifying fibroma, psammomatoid bodies, intracranial extension

## Abstract

Juvenile psammomatoid ossifying fibroma (JPOF) is a type of fibro-osseous lesion in which normal bone is replaced by fibrous tissue. Most commonly, it involves the nasal sinuses or orbital bone. It is very important to differentiate it from other fibro-osseous lesions because these lesions have overlapping clinical features. Even though it is a benign condition, it is locally aggressive and rarely extends intracranially. A complete surgical resection is the key to avoid recurrence. We are presenting a case of JPOF in a 16-year-old boy with a large tumor involving the ethmoidal sinuses with large intracranial component.

## Introduction

Juvenile ossifying fibroma is an uncommon benign condition. It is a type of fibro-osseous lesion characterized by a process leading to normal bone being replaced by fibrous tissue which contains different amounts of mineralized material. 1 It has rapid, expansive growth that affects children and adolescents. 2 Histologically, it shows two distinct patterns: juvenile trabecular ossifying fibroma (JTOF), which most commonly involves the maxilla in the preadolescent group (8–12 years), and juvenile psammomatoid ossifying fibroma (JPOF), usually originating from nasal sinuses or orbital bone in young adults (16–30 years). 3 We present a rare case of a 16-year-old male with juvenile psammomatoid ossifying fibroma of the ethmoidal sinus, extending into the anterior cranial cavity.

## Case Report

**Fig. 1:**
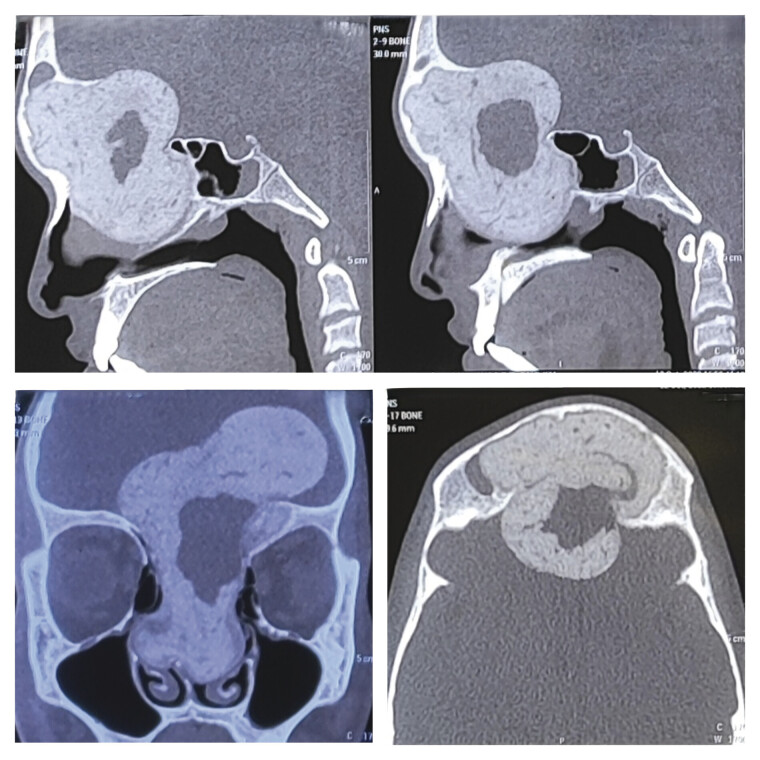
Computed tomography (CT PNS) clockwise—sagittal, sagittal, axial, and coronal images showing a well-defined, multilobulated, heterogeneously hyperdense lesion involving the ethmoid sinuses, frontal sinus almost entirely, also extending into the cranial cavity into the anterior cranial fossa. The lesion also has a hypodense cystic component in its center. PNS, para nasal sinuses.

A 16-year-old boy presented with a history of swelling over the forehead, moreover the right eyebrow, for 6 months, which was gradually increasing in size. He also complained of nasal congestion with preserved olfactory sensation for 2 months. On examination, the patient was conscious, oriented to the time and place, and had no motor or sensory deficit. There was no evidence of papilledema on fundus examination. A computed tomography (CT) brain scan showed a well-defined, hyperdense space-occupying lesion in the fronto-ethmoidal region with bone window showing a hypodense lesion eroding the frontal sinus ( Fig. 1 ). On further evaluation with contrast MRI of the brain, the tumor was seen to involve the ethmoid air cells and nasal cavity, superiorly extending to the anterior cranial fossa causing mass effect on the left frontal lobe.

**Fig. 2:**
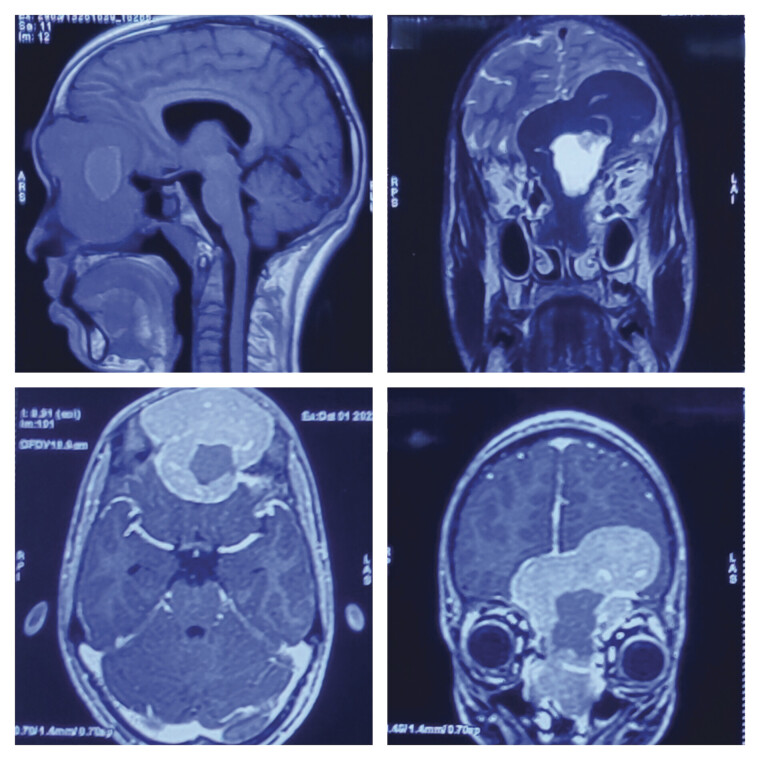
Preoperative magnetic resonance imaging (MRI) of brain. Clockwise—T1-weighted sagittal, T2-weighted coronal, T1-weighted post-contrast coronal, and T1-weighted post-contrast axial images showing well-encapsulated, multilobulated, heterogeneous lesion having a cystic component inside, T1 hypointense, T2 hyperintense with post-contrast enhancement filling the entire frontal sinus, extending into the nasal cavities and also extending superiorly into the anterior cranial fossa, compressing the frontal lobe and indenting it.

**Fig. 3:**
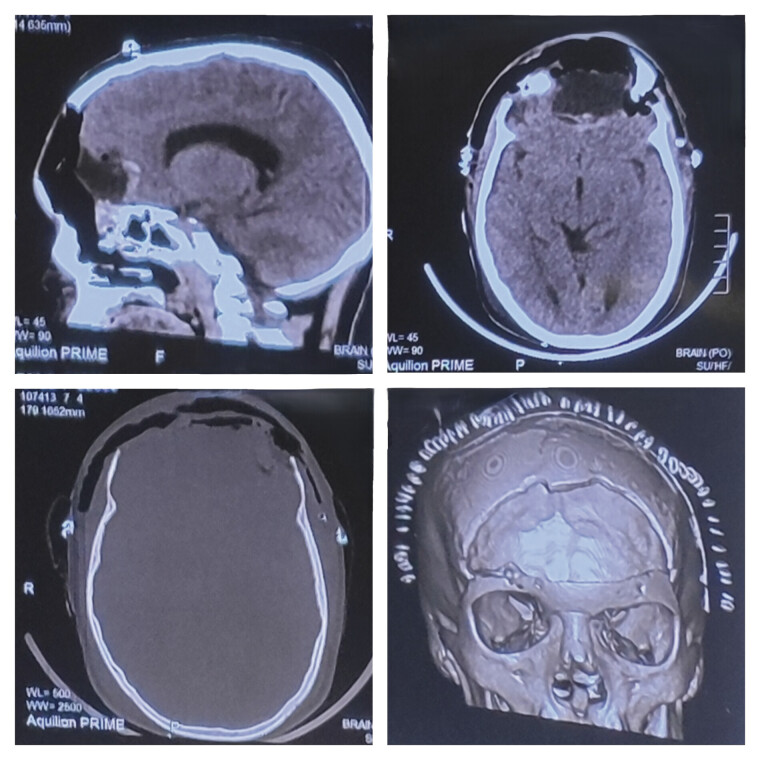
Postoperative computed tomography (CT) scan. Clockwise—sagittal, axial, three-dimensional reconstruction, and bone window axial images showing near-complete excision of the lesion with a bony defect in bilateral frontal region post bifrontal craniotomy.

**Fig. 4:**
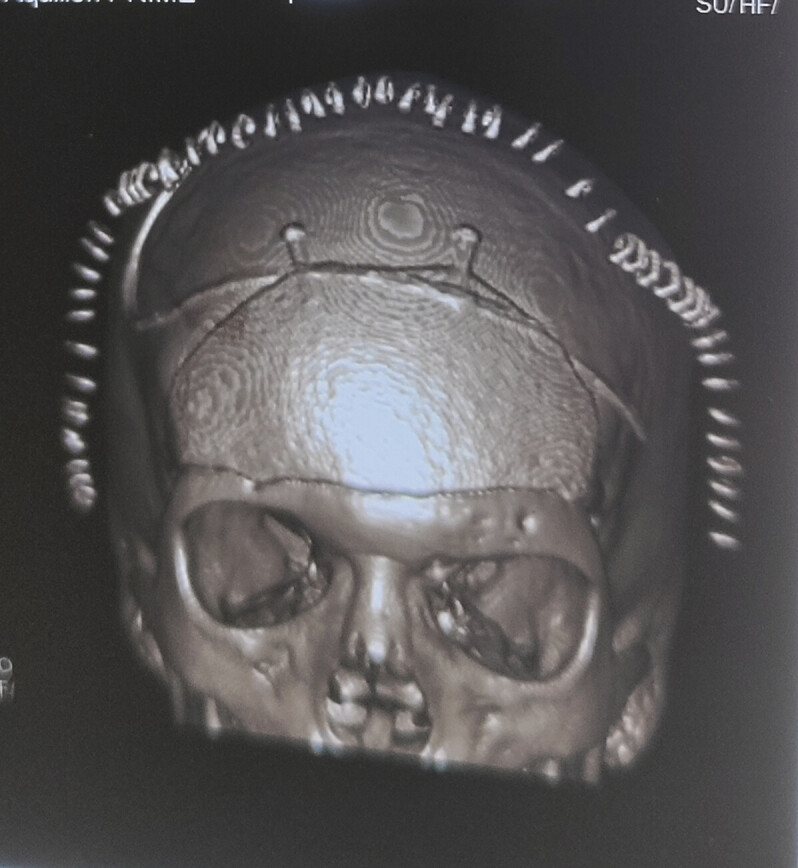
Postoperative computed tomography (CT) of brain three-dimensional reconstruction image after titanium precontoured implant cranioplasty, showing complete closure of the previous craniectomy defect.

**Fig. 5:**
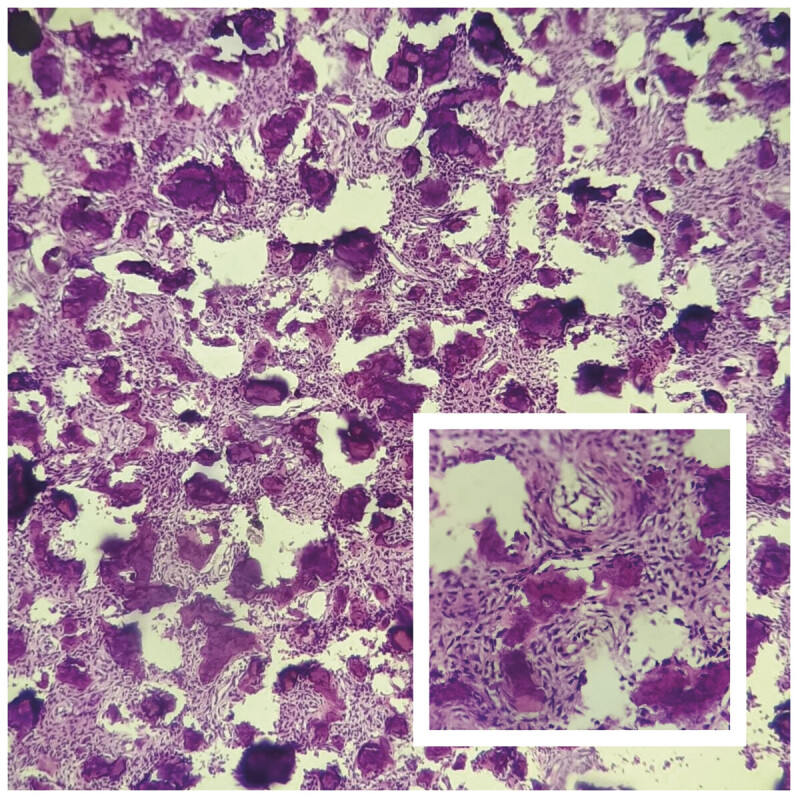
Histopathology of the excised specimen showing oval to stellate shaped cells in a vascularized collagenized stroma background interspersed with numerous lobules of calcification of varying sizes and shapes.

**Fig. 6:**
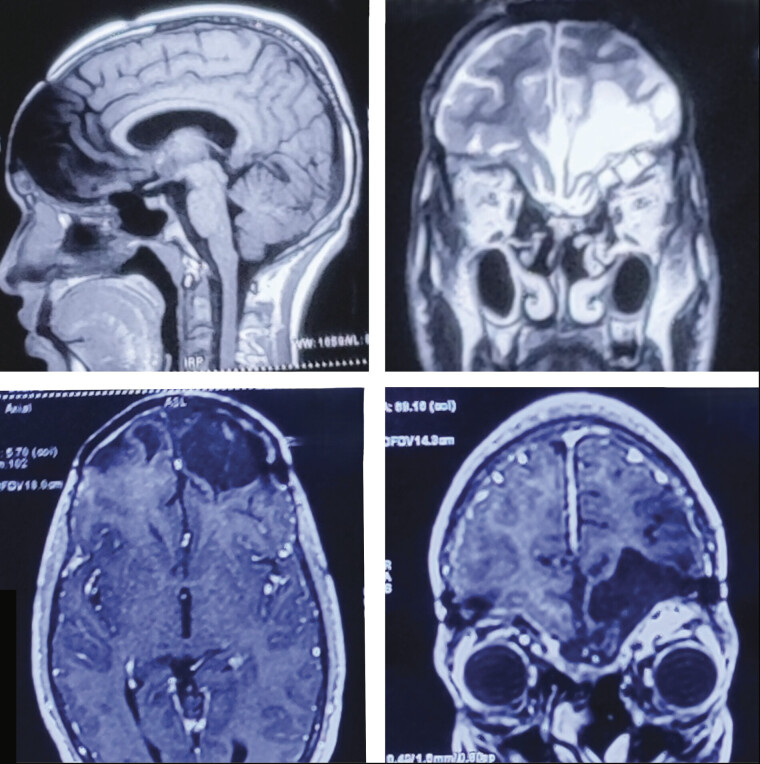
Magnetic resonance imaging (MRI) of brain at 1-year follow-up. Clockwise—T1-weighted sagittal, T2-weighted coronal, T1-weighted post-contrast coronal, and T1-weighted post-contrast axial images showing gliosis/encephalomalacial changes in bilateral basifrontal and left high frontal region with no evidence of any recurrence or any contrast enhancement.

Further, it was displacing orbit with heterogeneously altered signal intensity on T1 and T2 with intratumoral and peritumoral cysts with moderate post-contrast enhancement ( Fig. 2 ). The patient underwent a bi-frontal craniotomy with gross total excision of the tumor ( Fig. 3 ). Intraoperatively, the tumor was hard in consistency with involvement of the frontal sinus, frontal bone, and basifrontal dura and was extending inferiorly to involve the ethmoidal sinuses. Reconstruction of the skull base was done with pericranial grafts and fat. As the tumor involved the frontal bone it was discarded. The bony defect was repaired after 4 months using a titanium precontoured flap ( Fig. 4 ). The patient made a good recovery and was discharged. The final histopathology report confirmed psammomatoid ossifying fibroma ( Fig. 5 ). The patient was followed up at 1-year interval and MRI suggested no recurrence or residual tumor ( Fig. 6 ).

## Discussion

Fibro-osseous lesions are a group of benign lesions involving maxillofacial bones and paranasal sinuses. Fibrous dysplasia, ossifying fibroma, and osseous dysplasia are different categories of this group. These categories show more or less similar histopathological features. 4 According to the 2017 WHO classification of head and neck tumor ossifying fibroma, it is classified as cemento ossifying fibroma (COF), juvenile trabecular ossifying fibroma (JTOF), and juvenile psammomatoid ossifying fibroma (JPOF). 2

JPOF is difficult to differentiate from other fibro-osseous lesions because of overlapping features. The diagnosis of JPOF depends on clinical symptoms, radiological features, and pathological findings. 5 These patients with JPOF have symptoms like headaches, nasal congestion, exophthalmos, visual disturbances, and recurrent sinusitis. 6

Imaging studies like CT and MRI are used to distinguish JPOF from other fibro-osseous lesions and determine invasion into surrounding structures. 4 A well-circumscribed, multiloculated, expansile mass with thick wall bone density on CT scan and MRI isointense in T1 images, iso- to hypointense in T2 images, and post-contrast enhancement are suggestive of JPOF. 7 Important features that will differentiate JPOF from other fibro-osseous lesions by radiological investigations are the identification of peripheral margins, ground glass lesions, and tumor locations. 8

In our case, CT scan showed an ethmoidal nasal sinus mass with a ground glass appearance and bony erosion extending into the anterior cranial fossa. MRI suggested post-contrast enhancement with intra-tumoral cysts.

Pathological features of JPOF include a firm to hard, well-defined border, and histologically, it shows benign fibro-osseous proliferation composed of bony spicules and spherules mixed with a fibrous stroma. The main characteristic feature in histology is the presence of mineralized or calcified psammoatoid bodies or ossicles. 5

JPOF can be confused with primary extracranial meningioma which involves paranasal sinuses. 9 The characteristics that differentiate JPOF from meningioma are the immune reactivity of EMA and vimentin in meningioma and histomorphology of psammomatoid bodies of JPOF such as the presence of calcified spherules. 10 In meningioma psammomatoid bodies are distributed irregularly but in JPOF they are homogenously distributed. 10

In this condition, complete excision of the tumor is the treatment of choice because, even though it is a benign condition, it is locally aggressive. The recurrence rate of this condition after resection is between 30 and 56%. The main reason for recurrence is an incomplete resection. 11 The location and extension of the tumor are the main factors in deciding the surgical strategy—a total intracranial approach vis-à-vis a combined subfrontal and nasal approach. 12 In our case we selected a complete intracranial subfrontal approach because a large component of the tumor was intracranial with a smaller ethmoidal sinus component. 13

## Conclusion

JPOF is rare and typically presents in young age groups. It is a benign condition but locally invasive to surrounding structures and may extend intracranially. It is important to differentiate it from other fibro-osseous lesions because of overlapping clinical symptoms. A complete understanding of JPOF is important for increasing diagnostic accuracy, deciding on an accurate treatment plan, and improving prognosis.
